# Identification of Cognitive Vulnerability in Older Surgical Patients: A Retrospective Audit of Admission Screening

**DOI:** 10.1192/bjo.2026.11804

**Published:** 2026-06-30

**Authors:** Hugh O’Hare, Rachael Elliott, Nehal Shah

**Affiliations:** 1 Sheffield Teaching Hospitals, Sheffield, United Kingdom; 2 Essex Partnership University NHS Foundation Trust, Colchester, United Kingdom

## Abstract

**Aims:**

Cognitive impairment is common in older adults, yet many cases of dementia remain undiagnosed on hospital admission. Cognitive impairment is associated with increased risk of delirium, long-term cognitive decline, and adverse outcomes in hospitalised patients, particularly in surgical patients. Early identification may support recognition of undiagnosed dementia, help prevent delirium or further cognitive deterioration, inform capacity and consent assessments, and enable targeted psychiatric interventions. Within the acute surgical pathway, we observed inconsistent completion of a locally embedded cognitive screening section in admission clerking booklets. We therefore audited compliance with a trust standard that all patients aged ≥60 years should have a cognitive screen completed on presentation to the surgical assessment unit.

**Methods:**

The audit aimed to assess completion of cognitive screening on surgical admission and explore its association with operative intervention. 85 general surgical clerking booklets for patients aged ≥60 years admitted between February and July 2024 were reviewed. Data collected included documentation of cognitive screening, the presence of a known diagnosed cognitive impairment and progression to operative intervention within seven days of admission. Absence of clerking documentation was recorded where applicable. Data was analysed descriptively.

**Results:**

Of 85 surgical admissions aged ≥60 years, cognitive screening was documented in thirty-eight patients (45%). Thirty-seven patients (44%) had no documented cognitive screen, and nine patients (11%) had missing clerking documentation.

Of those with a documented screen, nineteen (50%) underwent an operative procedure within seven days, compared with eight of thirty-seven patients (22%) without a documented screen.

Three patients had pre-existing dementia. Cognitive screening was completed incorrectly in one case and classed as cognitive screen not documented.

**Conclusion:**

Low completion rates of cognitive screening on admission were observed, including among patients who subsequently underwent surgery. As data collection was limited to initial admission clerking booklets, some screens may have been completed onlater assessments, or conduced without documentation, potentially underestimating overall completion. The focus of this audit was assessment at point of admission. While patients proceeding to surgery would have undergone formal capacity assessment and consent prior to the intervention, admission screening serves a broader purpose. Guidance such as NICE delirium recommendations emphasise early identification of patients at risk of cognitive change. Further work is needed to explore barriers to completion, support targeted education, and ensure at risk patients of cognitive decline receive appropriate attention and multidisciplinary input.

